# Can behavioural nudges promote reduced-salt dish orders on meal delivery apps?

**DOI:** 10.1016/j.puhe.2024.12.028

**Published:** 2025-05

**Authors:** Wenyue Li, Chao Song, Ying Cui, Yue Ma, Beisi Li, Zhongdan Chen, Paige Snider, Ying Long, Ailing Liu, Gauden Galea

**Affiliations:** a School of Architecture, Tsinghua University, Beijing, China; b School of Architecture, Harbin Institute of Technology, Shenzhen, China; c National Institute for Nutrition and Health, Chinese Center for Disease Control and Prevention, Beijing, China; d World Health Organization Representative Office in China, Beijing, China

**Keywords:** Salt reduction, Nudging, Choice architecture, Meal delivery app, China

## Abstract

**Objectives:**

This study aimed to evaluate the effectiveness of educational health message and menu default options on a Meal Delivery App (MDA) in nudging consumers towards reduced-salt options in restaurants of China.

**Study design:**

We conducted a nudge-based intervention trial on an MDA named ELEME in China.

**Methods:**

Along with a control group, five intervention groups were formed utilizing different combinations of the three nudging treatments, including setting up a salt submenu for “reduced salt” default, a salt submenu for “regular salt” default, and a conventional educational health message on the ordering page. We recruited 903 restaurants from cities across different geographic regions and assigned them into either the control group or one of the five intervention groups.

**Results:**

After analyzing 870,942 meal orders, the results showed that the inclusion of a health message on the restaurants' ordering page was not effective to influence consumers to choose reduced-salt dishes (P > 0.1). A salt submenu that explicitly asked for consumers' preference for “reduced salt” or “regular salt” significantly increased consumers’ likelihoods of choosing reduced-salt dishes in the unadjusted model (P < 0.1).

**Conclusions:**

Applying choice architecture changes as nudge-based interventions on MDAs were proven to be effective to promote consumers to order reduced salt dishes, and we consider these findings to have real-world implications for policymakers, researchers, and the private sectors.

## 1 Introduction

Emerging research has shown that changing the food environment through nudging may help individuals make healthier eating choices.^1^, ^2^, ^3^ Traditionally, providing health information and educational or health literacy campaigns were the most common approaches for promoting healthy eating. However, recent studies found that decision structure nudges perform significantly better than educational and information campaigns.^4^ Studies have already applied nudge-based interventions to coax consumers to choose healthier eating options, such as making healthy options more prominent in menu design and measuring their outcomes by energy, fat, sugar, and sodium or salt intake.^5^, ^6^, ^7^ In the existing literature, most studies of food ordering nudging have been conducted on online food ordering platforms for specific settings, such as workplaces and school canteens,^8^, ^9^, ^10^ with some studies being conducted in virtual environments such as online surveys.^11^, ^12^, ^13^ None of them were conducted in a real-world environment nor on commercial food ordering platforms that serve the general public, which rendered their findings less powerful in providing recommendations for real-world practice. Therefore, the findings of these studies are not representative at the population level.

Nowadays, people across the world tend to consume more foods that are high in salt or sodium, which potentially increases their risk of developing hypertension.^14^ In China, 27.5 % of people aged 18 years and older are hypertensive, among whom only 11.0 % have their hypertension controlled.^15^ Evidence has also shown that reducing salt intake can significantly reduce blood pressure in adults with or without hypertension.^16^, ^17^, ^18^, ^19^, ^20^, ^21^, ^22^ Promoting strategies and actions aimed at reducing salt intake has been the target of the world.^23^^,^^24^ The report on the nutrition and chronic disease status of Chinese residents in 2020 showed that Chinese adults on average consume 9.3 g of salt a day from home cooking,^15^ well over the recommended 5 g per day by the World Health Organization (WHO) and the Chinese Dietary Guidelines.^25^^,^^26^ With the development of the economy and urbanization in China, ordering meal deliveries has become increasingly popular among Chinese residents.^27^^,^^28^ Recent evidence from studies in many countries has indicated that takeaway meals are often unhealthy, with their salt content exceeding the dietary recommendations and that of homemade meals.^29^, ^30^, ^31^, ^32^, ^33^ However, food ordering platforms typically provide little to no dietary information to guide consumer decisions, and no salt reduction interventions have been conducted in China targeting these platforms.

This study aimed to evaluate the effectiveness of information and menu default options on a Meal Delivery App (MDA) in nudging consumers towards reduced-salt options in restaurants of China.

We designed two different kinds of nudging interventions: educational information (reduced salt messages) as the decision information nudge, and using submenu with default on either “reduced salt” or “regular salt” as the decision structure nudge.^34^^,^^35^ Based on an intervention trial in real-world environment, we examined and compared the effects of these nudging interventions and their combinations.

## 2 Methods

### 2.1 Experiment design

**Fig. 1:**
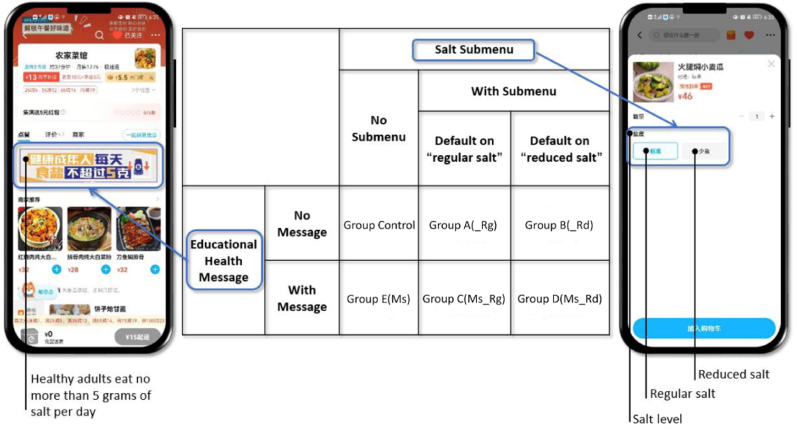
Design of groups and settings on the meal delivery app. Note: “Ms” stands for “with health information message”, “Rg” stands for ‘with salt submenu and default on “regular salt”’, and “Rd” stands for ‘with salt submenu and default on “reduced salt”’

We conducted a nudge-based intervention trial on an MDA in China to test the effectiveness of changing choice architecture in prompting consumers to choose reduced-salt options (Fig. 1). This study employed a 2 × 3 factorial design with two factors: information (yes/no) and submenu design (no, yes + default on “regular salt”, and yes + default on “reduced salt”). In specific, the intervention involved three treatments, including setting up a salt submenu for “reduced salt” and “regular salt” options; setting up a salt submenu defaulting on either “reduced salt” or “regular salt” options; and adding an educational health message “healthy adults eat no more than 5 g of salt per day” on the top of the ordering page. As shown in Fig. 1, five intervention groups, namely group A(_Rg), B (_Rd), C (Ms_Rg), D (Ms_Rd) and E (Ms), were formed utilizing different combinations of the three nudging treatments, along with a control group. For all the restaurants that did not offer the salt submenu, whether in the control group or in the intervention groups, according to the design of the MDA, consumers could request reduced salt for their dishes by leaving a message in the comment box (with a prompt “Please fill in the taste preference”) before placing the orders.

### 2.2 Restaurant recruitment and procedures

The study was conducted on ELEME, a major MDA in China. Restaurant recruitment took place from February to May 2021. Participating restaurants were instructed to adjust their restaurant interface on the MDA accordingly. We recruited restaurants already operating on the MDA and assigned them into six groups, including the five intervention groups and the control group.

We employed two methods to recruit restaurants on a voluntary basis from tier-1 and tier-2 cities across different geographic regions of China. For the first batch, an open invitation was sent by the MDA to restaurants in Beijing, Changsha, Guangzhou, Hangzhou, Xi'an, Shenyang, and Chengdu, resulting in 1937 sign-ups. For the second batch, targeted invitation was made through the research team's existing relationships with restaurants in Chengdu, Taizhou and Taiyuan, as well as a restaurant chain with branches across different cities in China, resulting in 494 sign-ups.

After recruitment, restaurants were randomly assigned to either the control group or one of the five intervention groups (as shown in Fig. 1), with each group comprising 405 to 406 restaurants. Restaurants in the intervention groups were required to select 1–3 of their most popular dishes for setting up salt submenus, if they were assigned with submenu treatment. However, only restaurants in Beijing, Shenyang, Taizhou, Chengdu and the restaurant chain demonstrated a high degree of cooperation in the setup process. Restaurants in other cities did not participate in the setup process and were excluded from the study.

Participating restaurants had not previously set up a salt submenu or an educational health message. Between signing up and the intervention period (July to August 2021), restaurants in the intervention groups were encouraged and instructed to adjust their restaurant interface on the MDA. To monitor and maintain compliance, weekly manual check-ups were conducted and recorded. Restaurants that did not maintain the assigned interventions during the intervention period were excluded from the study. Additionally, we created online groups with restaurant owners and managers to share free materials supporting salt reduction in restaurant settings and to provide guidance and supervision as needed.

### 2.3 Participating restaurants

Even the cities or the chain with good cooperation, not all restaurants fully adhered to the intervention setup requirements. By July 1, 2021, only some restaurants had correctly implemented the assigned intervention settings as designed, while many either did not set them up or discontinued early. A total of 131 restaurants maintained the correct intervention setup throughout the entire intervention period, while 772 restaurants signed up for the study but did not implement any intervention settings. For data analysis, the 131 compliant restaurants were categorized as intervention groups according to their settings, and the 772 non-compliant restaurants as the control group.

Table 1 outlines the characteristics of the participating restaurants and the number of orders generated during the intervention period. In total, 870,942 meal orders were placed through all participating restaurants, of which 256,726 orders came from the five intervention groups.

**Table 1:** Restaurant characteristics and the number of meal orders made on the MDA [N (%)].

Characteristics		Number of restaurants	Number of meal orders
Restaurant Location (City)	Chengdu	32 (3.5)	38,678 (4.4)
	Beijing	348 (38.5)	413,550 (47.5)
	Taizhou	321 (35.5)	182,001 (20.9)
	Shenyang	175 (19.4)	162,316 (18.6)
	Others^a^	27 (3.0)	74,379 (8.5)
Cuisine Category	Chinese cuisine	187 (20.7)	24,605 (2.8)
	fast food	479 (53.0)	384,442 (44.1)
	Others	237 (26.2)	461,877 (53.0)
Intervention Group	A(_Rg)	58 (6.4)	155,148 (17.8)
	B(_Rd)	16 (1.8)	29,065 (3.3)
	C(Ms_Rg)	14 (1.55)	16,946 (1.9)
	D(Ms_Rd)	6 (0.7)	7300 (0.8)
	E(Ms)	37 (4.1)	48,267 (5.5)
Control Group		772 (85.5)	614,216 (70.5)
Total		903	870,942

a The restaurant chain in cities other than Chengdu, Beijing, Taizhou, and Shenyang.

### 2.4 Data on reduced-salt dishes and orders

Data generated during the intervention period was extracted from the MDA's operation data and provided in Excel format anonymously (detailed in Appendix A). For participating restaurants in the salt submenu intervention groups, dishes ordered through the submenu and with the “reduced salt” option selected were counted as reduced-salt dishes.

For intervention group E (Ms) and the control group, those with consumer comments identified by keywords or phrases related to reducing the amount of salt contained in the ordered meal were regarded as reduced-salt orders. The number of reduced-salt orders was the number of orders with reduced-salt comments.

For intervention group A(_Rg), B (_Rd), C (Ms_Rg), and D (Ms_Rd), which contained a salt submenu, consumers could ask for salt reduction in dishes either by leaving a reduced-salt request in the comment box or by selecting the reduced-salt option on the submenu. Once an order included one reduced-salt dish or with a reduced-salt comment, it was considered a reduced-salt order. The total number of reduced-salt orders generated through intervention group A(_Rg), B (_Rd), C (Ms_Rg), and D (Ms_Rd) was a sum of reduced-salt orders made through consumer comments and reduced-salt orders made through the salt submenu. However, for reduced-salt dishes, the link with individual orders was not provided. Thus, the number of salt-reduced dishes had to be converted into the number of reduced salt orders to facilitate an overall comparison across intervention and control groups. According to the ELEME data, a meal order on average contained four items, including dishes and others (e.g., staple food and disposable tableware), so one order averagely had less than four reduced-salt dishes. By dividing the number of reduced-salt dishes made by the salt submenu by four, the number of orders made by selecting the reduced-salt option on the submenu was prone to being estimated. The estimation assumed all ordered items were reduced-salt dishes, regardless of the mixture nature of a meal order. For example, if a restaurant received 1000 reduced-salt dish orders through the salt submenu during the intervention period, we would estimate it as 250 reduced-salt takeaway orders for subsequent data analysis.

### 2.5 Statistical analysis

The statistical software STATA version 17 was used for data analysis. All the P values indicated the two-sided probabilities, and P < 0.1 was considered significant.^36^^,^^37^

### 2.6 Intervention effectiveness

Multiple linear regression models were used to test the intervention effectiveness by comparing the reduced-salt orders of restaurants in different intervention groups, with or without adjustment for covariates including restaurant location, cuisine category, and intervention category. The variable of restaurant location was classified as Shenyang, Beijing, Taizhou, Chengdu, and other cities (the branches of the restaurant chain located in cities other than the above four were also regarded as other cities). The restaurants signed up from the cities of Changsha, Guangzhou, Hangzhou, Xi'an, and Taiyuan were excluded from analysis due to the limited number of participating restaurants from these cities. The cuisine category included Chinese cuisine, fast food, and others, according to the self-labeling of each restaurant.

## 3 Results

### 3.1 Effectiveness of the educational health message and salt submenu

There was no significant difference in the number of reduced-salt orders made on the MDA between Group Control and Group E(Ms) in both univariate and adjusted models (Table 2).

**Table 2:** The salt reduction effect of the educational health message and salt submenu on the MDA.

	Coefficient
Effectiveness of the educational health message	
Univariant	
Group Control	1.0 (Ref)
Group E(Ms)	1.3
Adjusted^a^	
Group Control	1.0 (Ref)
Group E(Ms)	−0.7
Effectiveness of the salt submenu	
Univariant	
Group Control	1.0 (Ref)
Group A(_Rg) and Group B(_Rd)	9.3∗∗
Adjusted^a^	
Group Control	1.0 (Ref)
Group A(_Rg) and Group B(_Rd)	2.9∗

∗P < 0.1, ∗∗P < 0.05.

a Adjusted for restaurant location (city), cuisine category and intervention category.

The effectiveness of the salt submenu itself was significant in increasing the number of reduced-salt orders when the orders of Group A(_Rg) and Group B(_Rd), the intervention groups with the salt submenu, were compared with Group Control. The unadjusted salt-reduction effect of the salt submenu was greater than that of no intervention (coefficient: 9.3; P < 0.1). With an adjustment for covariates about the restaurants’ located cities and their cuisine categories, the effect of the salt submenu became smaller but remained significant (coefficient: 2.9; P < 0.1).

### 3.2 Effectiveness of different interventions

Table 3 shows the salt-reduction effect of different interventions. The salt submenu intervention with the default on either “regular salt” (Group A(_Rg)) or “reduced salt” (Group B(_Rd)), and the dual interventions of the educational health message and the salt submenu with default on “regular salt” (Group C(Ms_Rg)) showed significant effects (P < 0.1) on generating reduced-salt orders, in both the univariate and adjusted models. But the greatest effect was observed in the salt submenu intervention group with the default on “reduced salt” (Group B(_Rd)), which had the largest coefficient.

**Table 3:** The salt reduction effect of different interventions (educational health message, salt submenu, and educational health message combined with salt submenu) on the MDA.

	Coefficient
Univariant	
Group A(_Rg)	24.2∗∗∗
Group B(_Rd)	54.3∗∗∗
Group C(Ms_Rg)	33.6∗∗∗
Group D(Ms_Rd)	10.9
Group E(Ms)	1.3
Adjusted^a^	
Group A(_Rg)	23.5∗∗∗
Group B(_Rd)	49.5∗∗∗
Group C(Ms_Rg)	30.6∗∗∗
Group D(Ms_Rd)	9.8
Group E(Ms)	−1.3

∗∗∗P < 0.01.

a Adjusted for restaurant location (city), cuisine category and intervention category.

The combined intervention of the educational health message and the salt submenu with the default on “reduced salt” (Group D(Ms_Rd)) also had some effect on promoting more reduced salt orders, but not a significant effect. The effect of the educational health message on its own was not observed (coefficient: 1.3, P > 0.1).

## 4 Discussion

This is the first study to promote reduced-salt options for Chinese consumers through choice architecture changes on a commercial MDA. Although educational health messages have been used for decades to promote healthy eating behaviours in China,^38^ we found they had limited effect on increasing the choice of reduced salt meals on the MDA. This neutral effect is consistent with Harbers' review of 75 studies, which found that informational nudges had varied effects.^1^ In our study, we placed the educational health message at the top of the restaurants’ ordering page on the MDA. Consumers ordering from restaurants in the intervention groups without the salt submenu could only request a reduced-salt dish or meal by leaving a message in the comment box before placing the order. The ineffectiveness might be due to the extra effort required to act on the information, such as typing requests in the comment boxes. Even when combined with the salt submenu intervention, the educational health message did not enhance the salt reduction effect.

Unlike educational health messages, innovative approaches such as minor changes to choice architecture through decision structure nudges seemed more promising for promoting behavioural change among consumers. This study introduced two decision structure nudges by providing a salt submenu with explicit options over “reduced salt” and “regular salt” and by setting the default option as one of these. Although the results showed that both decision structure nudges were effective, when the default was on “reduced salt”, the effect was significantly greater in nudging consumers towards reduced-salt dishes. The decision structure nudges through default setting, or so-called “default nudges”, have shown good results in health-related domains and other areas of knowledge.^39^, ^40^, ^41^, ^42^ Among the studies, only one focused on single-use cutlery reduction on a real MDA.^43^ Our study introduced the MDA nudging method into the field of healthy food choice and provided new designs for decision information nudges and decision structure nudges at the dish level. However, ethical concerns about default nudges, frequently discussed in the literature,^44^^,^^45^ were also reflected in this study. While, the default “reduced salt” option proved to be the most effective intervention for nudging reduced-salt dish choice, fewer restaurants in these groups complied with the setting guidance compared to those with the default “regular salt” option. This result reflected restaurants' concerns that the default salt reduction settings might go against consumers’ desires. The effectiveness and propagability of different interventions need to be balanced.

Submenus are a setup already applied by the MDAs in China, which provide consumers with options over such things as portion size, and spice level, sugar level or ice level of a dish or drink. But this setup was rarely used for the level of salt by restaurants before this study. Data have shown that the total number of takeaways ordered through Chinese MDAs in 2021 was 20.3 billion, which represented 55.7 million orders per day on average.^46^ We therefore modeled the total number of reduced-salt orders that could be generated if the intervention was applied across all the MDAs in China, as an estimation of the effectiveness at population level. In our modeling, considering that 1.4% of the meal orders made through the intervention groups were reduced-salt orders, if the intervention was applied across all the MDAs in China, the number of reduced-salt orders could reach more than 779,000 per day, equivalent to 23.7 million in a month and 284.5 million across the year. If taking the intervention impact alone into consideration, an extra 445,000 reduced-salt orders could be generated across the MDA market in China every day, which would amount to 13.5 million extra reduced-salt orders every month, and 162.6 million extra reduced-salt orders every year. If the intervention was applied across all the MDAs in China, it would help generate an extra of over 160 million reduced-salt orders every year. According to the sodium content test conducted during the intervention period, the result of which is presented in another manuscript, the average sodium reduction between a reduced-salt version of a dish and a regular-salt version was 90 mg/100 g,^47^ with the average dish size being 575.6 g as found in a Chinese study.^26^, ^48^ Assuming every reduced-salt order would contain one reduced-salt dish at the minimum, that amounts to almost 150 tons of sodium, equivalent to 368 tons of salt being removed from Chinese MDA orders annually. This is a significant amount of salt that if taken out of Chinese people's diet, can greatly help reduce their risk of hypertension. The salt reduction effect of a salt submenu could be much larger if a “reduced salt” default submenu setup was provided for all dishes sold on the MDAs. We, therefore, encourage more attention and investment by policymakers, researchers, and the private sector in innovative approaches such as decision structure nudges as a supplement to conventional health education campaigns. The growing MDAs can be a new frontier for salt reduction and hypertension management in China.

Nevertheless, the current study does have some limitations. Firstly, the drop-out rate in each group differed, which might potentially have reduced the effectiveness of the intervention. For example, we only had six restaurants in Group D(Ms_Rd), and we observed significantly greater effect from the other salt submenu groups but not Group D(Ms_Rd). The small number of restaurants in Group D meant that the sample of orders cannot be sufficiently and evenly distributed across different location cities and cuisine categories, which may affect the reliability of the results. Secondly, the available data did not allow us to link reduced-salt dishes with the orders they belonged to, which prevented us from comparing intervention effectiveness at the order level using actual data. Instead, we estimated the number of reduced-salt orders of those with one or more reduced-salt dishes made through the salt submenu, which might be an underestimation, and this could potentially undervalue the effect significance of the salt submenu intervention. Thirdly, we did not collect the data on dish-related variables (e.g., dish category), which might also influence consumers’ reduced-salt choices. Fourthly, it was possible that the restaurants signed up for the study may have a higher willingness to promote salt reduction, or were more interested in salt reduction, as compared with those that did not participated in the study, which was also a limitation.

### 4.1 Conclusion

To promote consumers’ selection of reduced-salt dishes on MDAs, this study found that the inclusion of a salt submenu with explicit options over “reduced salt” and “regular salt” can boost the selection of reduced-salt dishes among consumers, and greater impact can be generated by defaulting on “reduced salt”. The findings enriched the theories of decision information and decision structure nudges, expanded the understanding of different interventions in improving dietary choices at population level, and implicated that introducing salt submenus to MDAs would be beneficial.

## Author statements

### Ethical approval

The study was reviewed and approved by Ethics Committee of the National Institute for Nutrition and Health of Chinese Center for Disease Control and Prevention (No. 2021–019), and WHO Western Pacific Regional Office Ethics Review Committee, with a trial registration number of ChiCTR2100047729. The research was done in line with ELEME user policies and regulatory policies around user privacy.

### Funding

This work was funded by the Resolve to Save Lives through their grant to the 10.13039/100004423World Health Organization China office to support efforts that aim to reduce salt intake among the Chinese population.

### Competing interests

None declared.

### Author contributions

WL: conceptualisation, data curation, formal analysis, methodology, software, original draft, review and editing. CS: conceptualisation, data curation, formal analysis, original draft, review and editing. YC, BL, ZC, PS and GG: conceptualisation, supervision, review and editing. MY: review and editing. YL and AL: resources, supervision, review and editing. All authors have read and approved the final manuscript.

### Data sharing plan

The data of consumer comments on the MDA are not publicly available.

### Disclaimer

The views expressed in this article are those of the authors alone and do not necessarily reflect the policies or views of the World Health Organization.

### Declaration of generative AI and AI-assisted technologies in the writing process

During the preparation of this work the authors used ChatGPT 4.0 in order to improve readability and language. After using this tool, the authors reviewed and edited the content as needed and take full responsibility for the content of the publication.

### Acknowledgments

The authors would like to thank all team members in this project, including following individuals from ELEME for their invaluable services to implement the project on their platform: Shuhan Zhang, Hong Miao and Xiyan Tian. The authors acknowledge the technical contributions made by Yizhen Gu and Shicheng Yu.
