# Supplementary material for: Can behavioural nudges promote reduced-salt dish orders on meal delivery apps?
Source: Public Health. 2025 May;242:250–5. doi: 10.1016/j.puhe.2024.12.028 (PMC12053955; doi:10.1016/j.puhe.2024.12.028)
Supplement: Multimedia component 1 [file mmc1.doc]

**Appendix A**

Data collected from the MDA included the following five sheets:

1. *Grouping Baseline Data*

Fields: time stage, shop ID, order number, longitude, latitude, score, shop name, address, type of cuisine, province name, city name, district(county) name

Date: 1st period, 1 July -- 31 August 2020; 2nd period, 1 July -- 31 August 2021; 3rd period, 1 September -- 31 December 2020, for each period

1. *Dishes Ordered by Submenu*

Fields: shop ID, commodity ID, commodity name, submenu choice, order quantity, province name, city name, district(county) name, shop name, date

Time: July 7, 2021 -- August 31, 2021, daily

*③ Submenu Default Setting*

Fields: shop ID, commodity name, the default setting for reduced-salt, options provided in the submenu, province name, city name, district(county) name, date

Time: July 7, 2021 -- August 31, 2021, daily

1. *Comments*

Fields: shop ID, reduced-salt comments, shop name, province name, city name, district(county) name

Time: March 1, 2020 - August 31, 2021, daily

1. *Shop Operation*

Fields: shop ID, order number, shop name, type of cuisine, province name, city name, district(county) name, week number

Time: March 1, 2020 - August 31, 2021, weekly
